# Significant acceleration of emergency response using smartphone geolocation data and a worldwide emergency call support system

**DOI:** 10.1371/journal.pone.0196336

**Published:** 2018-05-23

**Authors:** Michael Weinlich, Peter Kurz, Melissa B. Blau, Felix Walcher, Stefan Piatek

**Affiliations:** 1 University of Magdeburg, Department of Trauma Surgery, Magdeburg, Germany; 2 Hospital am Steinenberg Reutlingen, teaching facility affiliated with the University of Tübingen, Department of Trauma Surgery, Reutlingen, Germany; 3 University of Tubingen, Medical Center, Tubingen, Germany; National Yang-Ming University, TAIWAN

## Abstract

**Importance:**

When patients are disorientated or experience language barriers, it is impossible to activate the emergency response system. In these cases, the delay for receiving appropriate help can extend to several hours.

**Objectives:**

A worldwide emergency call support system (ECSS), including geolocation of modern smartphones (GPS, WLAN and LBS), was established referring to E911 and eCall systems. The system was tested for relevance in quickly forwarding abroad emergency calls to emergency medical services (EMS).

**Design:**

To verify that geolocation data from smartphones are exact enough to be used for emergency cases, the accuracy of GPS (global positioning system), Wi-Fi (wireless LAN network) and LBS (location based system) was tested in eleven different countries and compared to actual location. The main objective was analyzed by simulation of emergencies in different countries. The time delay in receiving help in unsuccessful emergency call cases by using the worldwide emergency call support system (ECSS) was measured.

**Results:**

GPS is the gold standard to locate patients with an average accuracy of 2.0 ± 3.3 m. Wi-Fi can be used within buildings with an accuracy of 7.0 ± 24.1 m. Using ECSS, the emergency call leads to a successful activation of EMS in 22.8 ± 10.8 min (Median 21 min). The use of a simple app with one button to touch did never cause any delay.

**Conclusions and relevance:**

The worldwide emergency call support system (ECSS) significantly improves the emergency response in cases of disorientated patients or language barriers. Under circumstances without ECSS, help can be delayed by 2 or more hours and might have relevant lifesaving effects. This is the first time that Wi-Fi geolocation could prove to be a useful improvement in emergencies to enhance GPS, especially within or close to buildings.

## Introduction

Time is one of the most important factors for the survival of emergency patients.[1–4] Plenty of studies have sufficiently proven that each link of the chain of survival has to be optimized.[5] This study focuses on a relevant problem concerning the activation of the emergency response system on a worldwide basis and a simple solution using international networks and smartphone technology.

All studies regarding activation of emergency response systems start their measurements when the call is received at the alarm center.[6–8] Any time delay between the triggering event and the successful activation of the emergency response system is difficult to measure. Therefore deviations are ignored. The time necessary to make an emergency call is considered to be very short.[9]

Especially when dealing with remote or internationally travelling patients, the time to activate the emergency response system abroad becomes relevant when patients are disorientated or experiencing language barriers. E.g. an American traveling in Shanghai, China, trying to call a Chinese emergency response center will be confronted with the Mandarin language and will have difficulties explaining his correct actual location in a case of an emergency. These cases have never before been scientifically analyzed and could lead to time delays of several hours.

This phenomenon of disorientation in reporting emergencies is a known problem for EMS. Cases with time delays of more than 3 hours were even excluded in relevant trauma studies.[8]

Nowadays mobile networks guarantee availability almost everywhere and provide communication in emergency situations to initiate an emergency call by the patient.[10] The use of "foreign" mobile networks is possible when dialing emergency numbers, like 911 or 112, which allows access to call service without being locked into a specific mobile network.[11]

Even though the call reaches the alarm center, the dispatchers are confronted with the inability of the caller to express the appropriate location. Even with excellent location knowledge of the dispatcher, it might take long to overcome the location unfamiliarity of the caller. As in many places the catchment areas of the alarm centers are becoming larger, resulting in a more and more demanding task.[12]

Several studies reveal communication problems in emergency situations.[13,14] These lead to unclear situations and finally to relevant delays in helping the patients.[15–17]

Since the introduction of smartphones, a wide range of applications have become available to support emergency calls. In 1996 the "Enhanced 911 (E911)" system was started in the United States, providing coordinates of intersections to the alarm center.[18] In Europe the "eCall" system has been initiated to inform alarm centers about car accidents including geolocation information.[19,20] A 4% reduction in mortality was calculated due to reduction of time to inform the alarm center and provision of geolocation data. As the eCall system is just been introduced, prospective studies are still missing.

The goal of the study is to demonstrate that, even if a patient is disoriented or experiences language barriers, it is possible to activate the local EMS within a short time, not exceeding one hour. Using the different tracking options, global positioning system (GPS), wireless LAN (Wi-Fi) (Wi-Fi is a defined subgroup of WLAN) and location based services (LBS) of the patient, the geolocation data should quickly and reliably be forwarded via an international network to the local alarm center in the country of the patient.

The following questions were answered in the study:

Can new technology optimize the time of activation of the emergency response system even in the cases of disorientation and language barriers?

Is the time delay of a worldwide emergency call support system (ECSS) short enough to still be considered helpful for the chain of survival?

Is Wi-Fi an appropriate worldwide enhancement to provide relevant location data when GPS is missing, e.g. within concrete buildings?

## Material and methods

### Accuracy of GPS, Wi-Fi and LBS

In order to measure the accuracy of GPS, Wi-Fi and LBS,[21] 232 tests in eleven different countries were performed. Volunteers in these countries described their current position and measured their GPS, Wi-Fi and LBS position.

LBS and Wi-Fi geolocation data were determined using the app "Network Info II" (Alexandros Schillings, Maidenhead, UK). For GPS geolocation, the app "GPS-Test" (Chartcross Limited, New Milton, UK) was used S1 Text and S1 Protocol. The actual position of the volunteer, in relation to surrounding landmarks, was manually determined in Google Maps. The primary outcome of this sub-study was to determine the accuracy and differences of locating with the different geolocation approaches.

### Smartphones in use for emergency call simulation

Android or Apple smartphones with Wi-Fi and GPS functionality were used to simulate emergency calls. SMS and e-mail communication were activated. For instant transfer of geolocation data, the SOS-Call.eu app (med con team GmbH, Germany), was deployed by the test persons (volunteers) on their smartphones.

**Fig 1:**
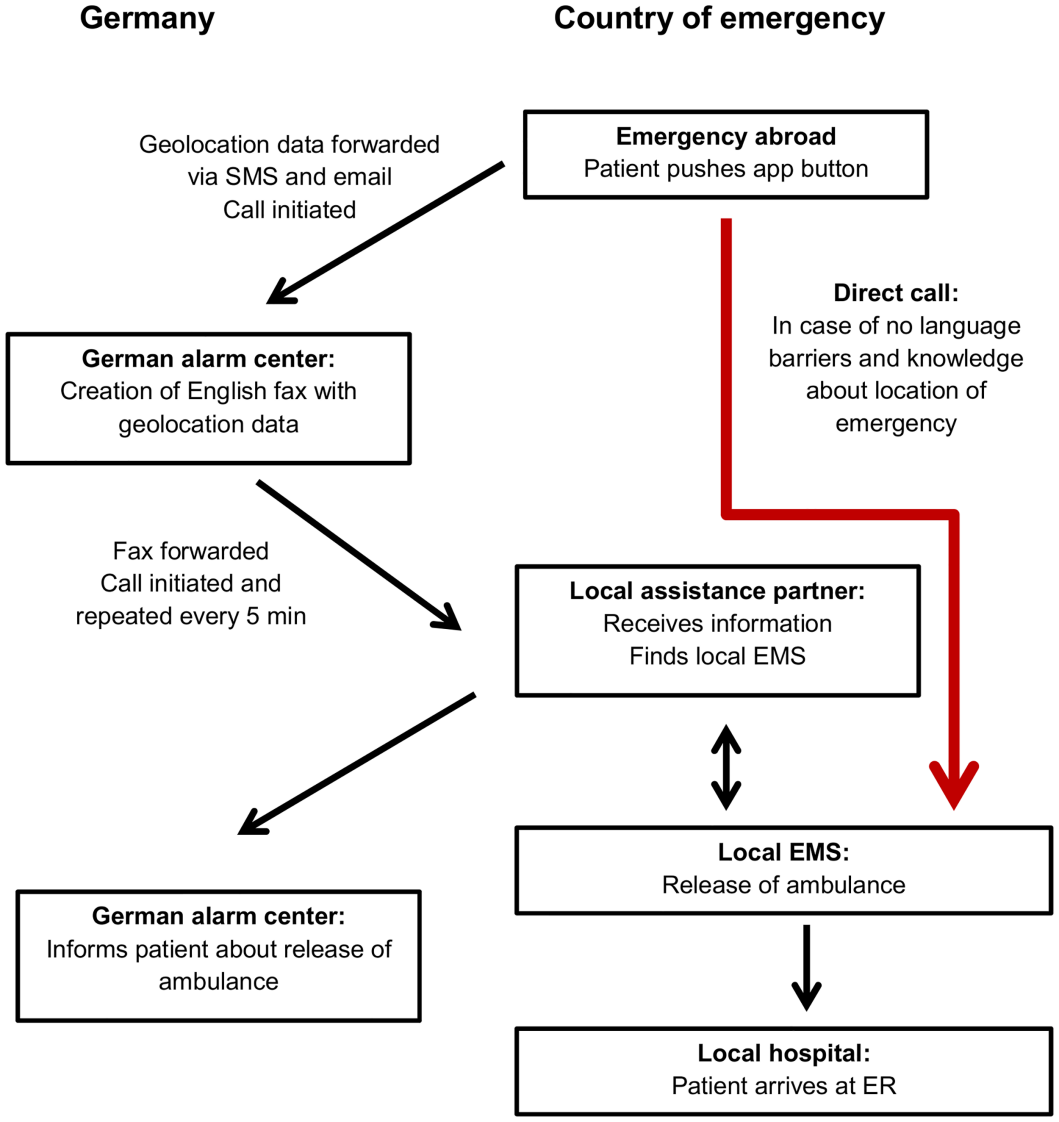
Process description of the emergency call support system (ECSS). In case of disorientation and language barriers, a direct call to the local EMS alarm center (red arrow) is not possible. Therefore the geolocation data have to be forwarded via SMS and e-mail to the German alarm center. A call was initiated to verify the emergency situation and geolocation data. Afterwards, a fax was generated and sent to the local assistance partner in the country of the patient. The local partner forwards the fax in local mother tongue to the local EMS alarm center. Finally, the patient was brought by EMS to the local hospital.

The app has just one red button and in case of an emergency the red button has to be pushed. Instantly the app reads the geolocation data present in the smartphone and immediately sent it in ASCII code both via SMS and e-mail to the German alarm center to be displayed within about 15 seconds on the monitor of the dispatcher. In addition, a call was automatically initiated by the app to connect the volunteer abroad to the German alarm center. The dispatcher was informed about the emergency and verified the received geolocation data with the caller (Fig 1).

### Transfer of emergency calls via international network to local EMS

Once all geolocation data is available and the kind of emergency is known, the dispatcher created a fax, including a relevant extract of the location of the patient out of Google Maps. The fax was forwarded to the local assistance partner in the country of the emergency. A local assistance partner is a company with a call center handling medical cases of insured patients or staff of corporate clients traveling abroad. The partner chose the appropriate EMS alarm center responsible for the emergency and forwarded the fax together with a call in the mother tongue of the country. Every 5 min the local assistance was reminded to provide a final confirmation until evidence was provided that the EMS was activated to help the patient (Fig 1).

### Simulation of international emergency calls

Since real emergency cases seldom happen in a given population, a simulation scenario was established. Approval was obtained from all involved parties prior to the simulation. The simulation scenario was just known to the test person in a specific country and the head of alarm centers in German and abroad. As the German alarm center was already dispatching real worldwide emergencies using the ECSS, the dispatcher was never aware if a real case or a simulation was present. The abroad local assistance partner’s staff was not informed about any simulations.

### Adaption of worldwide simulation to minimize harm to real EMS cases

Even in simulation cases, the above described process would automatically activate an ambulance car to drive to the test person’s location. A misuse of the EMS system had to be prevented. Therefore, during the research period, the standard process was altered and the local assistance partner was first asked to call the local EMS alarm center to gather the name of the emergency operator, the fax and telephone number. This information was first forwarded back to the German alarm center before an EMS was released.

As the head of operations of the German alarm center was always informed about incoming SMS and e-mails of the emergency calls, it was possible to stop the simulation before EMS was released. After the research period the process was returned to standard protocol.

### Reference group of failed emergency calls

To evaluate the effectivity of the support of the emergency call, a control group would be desirable. Unfortunately, cases of failed emergency calls and their delay in rescue time due to disorientation and language barriers are not reported or published as scientific data. Millions of participants over a long period of time would be necessary to obtain sufficient cases for comparison. Therefore, it was not possible to establish a simultaneous reference group to the emergency call support system. As a standard control group for comparison could not be achieved. To show the dimension of the problem, cases from press were randomly selected. All of the cases appearing in press have to be considered outliers.

Cases from press were selected by the following criteria: the person in an emergency situation had to be in a helpless situation and survived. Only cases were collected that were due to disorientation and language barriers (Table 1). The emergencies had to be in an area of high activity of EMS to provide a realistic comparison concerning the chance to be found and short transport times. Therefore only cases in Central Europe were chosen.

**Table 1:** Reference group of failed emergency calls. Emergencies with failed emergency calls due to disorientation and language barriers were collected from press. References of all cases are available.

Case	Year	Age of patient	Time from incident to arrival of help subtracted by average arrival time of EMS	Remarks
**1**	2012	27	10.5 h	
**2**	2012	adult	3.5 h	At sea
**3**	2009	60	1.5 h	Hypothermia
**4**	2010	24	0.5 h	Hypothermia
**5**	2008	51	0.5 h	
**6**	2012	78	5.5 h	Fall down
**7**	2012	17	0.5 h	
**8**	2011	78	1.5 h	Car accident
**9**	2011	56	0.5 h	
**10**	2012	79	6.5 h	Hypothermia
**11**	2009	20	2.5 h	Hypothermia
**12**	2012	48	1.5 h	Bicycle accident
**13**	2012	45	2.5 h	Car accident
**14**	2012	29	0.5 h	Bicycle accident
**15**	2012	70	3.5 h	
**16**	2011	66	7.5 h	Mountain accident
			Median 2.0 h	

As times reported in press sum up the time from the event until arrival in hospital, the average time for EMS to transport of a patient from the scene to hospital had to be subtracted. The average time for EMS to care for the patient and bring them from the scene to the hospital is about 30 min.[22]

### Pilot study

To determine the amount of simulation cases needed, n = 10 simulations using the worldwide emergency call support system application software (ECSS) were performed. For power calculation of the necessary number of patients in the simulation study, the assumption was made that the expected mean values have to lie within a standard deviation range of 10 min. The resulting sample size was n = 30.

## Results

### Accuracy of geolocation systems in comparison to different countries

**Fig 2:**
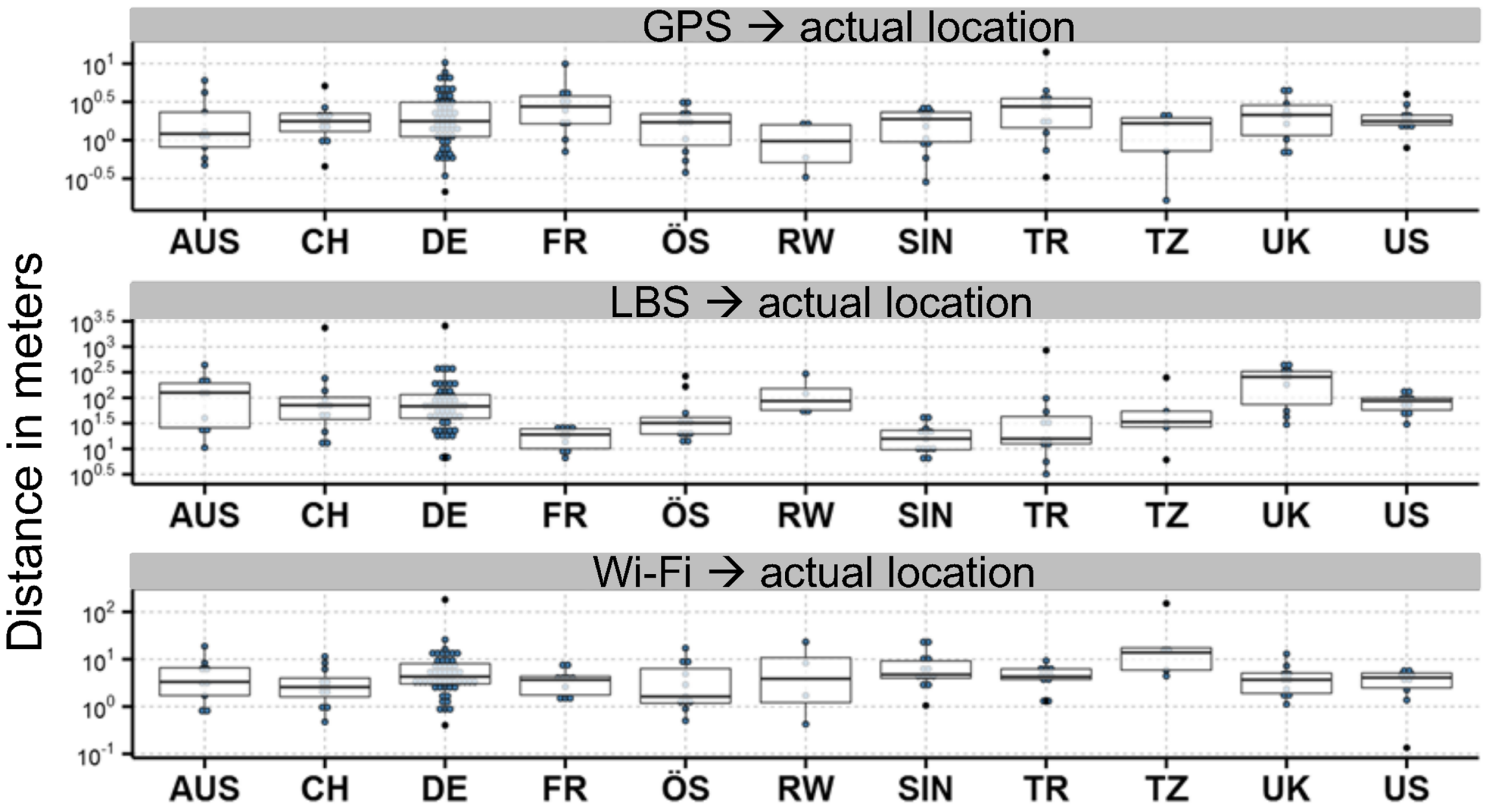
Comparison of accuracy of geolocation data in different countries are similar within the GPS, LBS and Wi-Fi groups) (n = 232). As differences between the accuracy of GPS, LBS and Wi-Fi are quite large, different scales had to be used between the 3 plots. No significant differences within one group of geo location systems could be detected between the different countries. AUS Australia, CH Switzerland, DE Germany, FR France, ÖS Austria, RW Rwanda, SIN Singapore, TR Turkey, TZ Tanzania, UK United Kingdom and US USA.

In eleven countries on different continents, GPS, LBS and Wi-Fi geolocation data were compared to the real position of the person S2 Dataset. Within the different geolocation measurement systems, no relevant differences between the countries were found. GPS accuracy was below 10 m almost everywhere, Wi-Fi had a similar range, but was not as accurate, with diversions up to 100 m. LBS divergence even exceeded 1 km with a maximum error in distance of up to 2.5 km (Fig 2).

### Average accuracy of GPS, LAN and LBS geolocation

The average worldwide accuracy of GPS is in the same range and at 2 m ± 3 m, very close to the actual location of the person. Wi-Fi accuracy was 7 m ± 24 m on a worldwide basis. LBS average was 214 m ± 321 m, but very different in each country. The difference between GPS/Wi-Fi (d_Cohen_ 0.292, 95% confidence interval (0.034–0.551)) was not relevant, whereas the difference between LBS and GPS/Wi-Fi (d_Cohen_ 0.955, 95% confidence interval (0.683–1.226; d_Cohen_ 0.659, 95% confidence interval (0.659–1.2)) was relevant.

### Reference group of failed emergency calls

The evaluation of the individual cases resulted in a time between accident and finding the injured or diseased persons subtracted by the average arrival time for EMS, with a median of 2.0 h.

### Simulation of worldwide emergency calls

In thirty-three cases worldwide emergency calls were simulated S2 Dataset. The mean time to activate the local EMS was 0.38 ± 0.18 h and the median 0.35 h or 21 min.

The group of failed emergency calls from press reveals a (median of 2.0 h). Even though the difference of 1.65 h or 1 hour 39 minis noted, it is just a descriptive comparison.

## Discussion

In Germany, approximately 17 million emergency calls are dispatched each year via the rescue services. In about 10% of the cases, i.e. 1.7 million emergency calls, the callers do not know their position. This phenomenon is not recorded in any statistics, but has always been known to employees of the rescue service. Such cases are for instance excluded in the statistics of the trauma registry.[1]

Missing knowledge of the exact localization leads to considerable time delays until the rescue service finds the patient. The significance of this phenomenon becomes clear when one considers the effects on the survival of patients. For emergency patients, time is a very important survival factor. In polytrauma it could be shown that under certain conditions a delay of 3 min to the final care increases the mortality by 1%.[4] Using this calculation of Clarke et al. mortality might be influenced by 10% for a 30 min delay.[4] Concerning other diseases, such as myocardial infarction or stroke [2], a delay caused for example by long search for the patient, leads to increase in mortality, too.

Calculations are being done on how many people’s lives can be saved via the E-Call in cars.[19] An estimate of about 2500 lives per year was mentioned. The annual traffic mortality on Germany’s roads was 3475 people in 2015. From a statistical point of view, a person is only for a maximum of 4% of the lifetime inside a car. Thus, an advanced system, like the ECSS, is desirable for the remaining 96% of time with the same intention of rapid delivery of help. Rough calculations reveal a life-saving benefit of ECSS, but further studies with high sample sizes have to follow to provide evidence.

GPS is the gold standard for geolocation. With its accuracy below 10 m it is useful for EMS to quickly find patients.[20] The study showed that today’s smartphones with integrated GPS are appropriate and fast enough to provide the necessary information. The main disadvantage of GPS is the necessity of unobstructed line of sight to the satellites. The study revealed that Wi-Fi geolocation can also be used to find patients. Wi-Fi-based geolocation requires an active internet connection, and is done by doing a Wi-Fi scan, noting the BSSIDs (the unique numeric hardware MAC addresses) of the Wi-Fi APs (wireless routers) in range, and sending that list of BSSIDs to a web service that looks up the known geo-coordinates of those APs, and reports back what the geo-coordinates must be, based on what APs the user is closest to. As Wi-Fi routers are usually located within buildings, patients within these buildings and in the vicinity (< 300 m) can be located. Other non-medical studies confirmed that the average distance to the actual location was less than 10 m.[21] As Wi-Fi routers provide the geolocation data without need of a login, all reachable Wi-Fi routers are automatically used to calculate the correct geolocation.

In a worldwide setting, both GPS and Wi-Fi provided sufficient accuracy to be used for EMS to find a patient as fast as possible. This is the first study to use Wi-Fi geolocation system for use in emergencies. As Wi-Fi can be used indoors too, it will be a useful addition to GPS.

LBS is too inaccurate to be used for EMS. It might have an additional impact on verifying validity of GPS and Wi-Fi data.

Grow et al. provided evidence that using the mother tongue is essential in describing an emergency situation and avoiding misunderstandings.[16] Even though this problem was minor in a US based alarm center, the time delay was significant. Not only the difficulty in describing the current location caused misunderstandings, but also the correct recognition by the dispatcher is essential.[23]

Unfortunately, neither a statistically correct control group could be developed, nor had it before been described in detail. The cases from the press at least provide amplitude of the known problem all over the world in a delay between the medical trigger and being able to call help to the appropriate location of the patient. The medium of 2.0 h to receive help due to disorientation or language barrier should better be seen as a range starting from 30 min up to more than 10 h that has influence on the survival ofpatients.

On a worldwide basis and most significantly in spacious areas,[24] the described emergency call support system (ECSS) will reduce the overall time from onset to final treatment. The average time to activate the emergency response system in case of disorientation or language barrier of 21 min (0.38 h) is longer than a successful communication to the local alarm center. Compared to the times from the scene to the ER that might exceed one hour in several parts of the world, it can be seen as an acceptable time delay.[12,20,24–27]

It is important to mention that the regular emergency call directly to the alarm center is still the shortest and most preferred way of activating the emergency system.[9] The used system has no active tracking, providing full data protection towards the patient. The only parameter that has to be known about the patient is the phone number.

On a worldwide basis, Wi-Fi enhanced GPS is especially suited for appropriate location of the patient in emergencies. Based on our results both systems should be used simultaneously for best geolocation of patients in the future.

Our attempt to optimize an emergency call in a numerically non-negligible population in emergencies (cases of disorientation and language barriers) using ECSS proved to be feasible and provides a significant effect. As these specific situations happen to thousands of patients every year, but are not well recorded, using ECSS will enlighten this area of emergency medicine.

## Supporting information

S1 Dataset Database of simulated cases. (XLSX)

S2 Dataset Database of determination of accuracy of GPS, Wi-Fi and LBS. (XLSX)

S1 Protocol English protocol to gather data of accuracy of GPS, Wi-Fi and LBS. (PDF)

S1 Text English instructions on how to determine accuracy of GPS, Wi-Fi and LBS. (PDF)

S2 Text Translated ethics commission statement. (PDF)

S3 Text Original German version of ethics commission statement. (PDF)
