# Supplementary material for: Significant acceleration of emergency response using smartphone geolocation data and a worldwide emergency call support system
Source: PLoS One. 2018 May 23;13(5):e0196336. doi: 10.1371/journal.pone.0196336 (PMC5965832; doi:10.1371/journal.pone.0196336)
Supplement: S1 Protocol — (PDF) [file pone.0196336.s003.pdf]

Name, First name: **Cell-Nr:** **Mail:** **Date:**

**Operator**	☐ Vodafone	☐ O2	☐

	**City/ Town**	**Out of town**	**Freeway**
**LBS**	☐	☐	☐
Location with national recognition (e.g. GER – Tuebingen, downtown			
Accuracy in meters			
Location Network Info II	Lat N:	Lon E:	
Address Network Info II			

**WI-FI**	☐	☐	☐
Location with national recognition			
Accuracy in meters			
Location Network Info II	Lat N:	Lon E:	
Address Network Info II			

**GPS**	☐	☐	☐
Location with national recognition			
Accuracy in meters			
Location GPS-Test	Lat N:	Lon E:	
Speed (km/ h):	Altitude (mtr):	Heading (degrees):	

Your real position	Description of your real position as accurate as possible
