# Supplementary material for: Significant acceleration of emergency response using smartphone geolocation data and a worldwide emergency call support system
Source: PLoS One. 2018 May 23;13(5):e0196336. doi: 10.1371/journal.pone.0196336 (PMC5965832; doi:10.1371/journal.pone.0196336)
Supplement: S1 Text — (PDF) [file pone.0196336.s004.pdf]

Before the test:

Required Software: Network Info II (Play Store → search for "Network Info ii")
GPS-Test by Chartcross (Play Store)

Please download and install the required software on your smartphone

Open GPS-Test →	Menu →	Settings →	Speed units→	Metric
			Altitude units →	Metric
Open Google-Maps→	Menu →	Settings →	Display →	Show Scale bar

Start your Smartphone, GPS and WI-FI should be turned off. Wait for updates and system check to be finished

Stay in one place during the test. Please do not walk around or do tests while driving

Tests:

Test LBS:

- Open Google-Maps and check, if the scale bar is shown
- Wait until the blue circle around your position remains constant
- Screenshot with scale→ the whole blue circle should be seen. Eventually make another screenshot after zooming to your position. LBS might have a radius up to 1,5 km or more

- Open Network Info II
- Open Tab „Location"
- Screenshot
- Fill in the protocol form

Test WI-FI:

- Turn on WI-FI
- Open Google-Maps
- Wait until the blue circle around your position remains constant. If WI-FI is available, the radius around your position should decline
- Screenshot with scale

- Open Network Info II
- Open Tab „Location"
- Screenshot (Careful: Note, that sometimes the old data of the LBS-Test are shown → If so, start Network info II again)
- Fill in the protocol form

Test GPS:

- Turn off WI-FI, turn on GPS
- Open Google-Maps
- Wait until the blue circle around your position remains constant
- Screenshot with scale

- Open GPS-Test → Write down "Accuracy", make another screenshot
- Open Tab „Map of the world" → Write down coordinates, make another Screenshot
- Open Tab "Speed/ Altitude/ Heading → Screenshot
- Fill in the protocol form

Your real position

- Mark your real position on a map (best: Google Maps)
- Eventually take some photos of a building or prominent place, which can be seen on a satellite image
- Send your screenshots and the protocol by mail to Peter.Kurz1984@web.de
- THANK YOU!!!!!!!!!!!!
