# Supplementary material for: Significant acceleration of emergency response using smartphone geolocation data and a worldwide emergency call support system
Source: PLoS One. 2018 May 23;13(5):e0196336. doi: 10.1371/journal.pone.0196336 (PMC5965832; doi:10.1371/journal.pone.0196336)
Supplement: S3 Text — (PDF) [file pone.0196336.s006.pdf]

UNIVERSITÄTSKLINIKUM
MAGDEBURG A.ö.R.

OTTO VON GUERICKE
UNIVERSITÄT
MAGDEBURG

TOP
NATIONALES
KRANKENHAUS
2017
FOCUS

Ethik-Kommission
der Otto-von-Guericke-Universität
an der Medizinischen Fakultät
und am Universitätsklinikum
Magdeburg A.ö.R.

Univ.-Prof. Dr. med. Christof Huth
Vorsitzender

Dr. med. Norbert Beck
Geschäftsführer

Telefon: +49 391 67-14314
Telefax: +49 391 67-14354
elektr.Fax: +49 391 67-290185
eMail: ethikkommission@ovgu.de

Ethik-Kommission, Medizinische Fakultat / Universitätsklinikum, Leipziger Str. 44 Haus 28, 39120 Magdeburg

Herrn PD Dr. med. S. Piatek
Universitätsklinik für Unfallchirurgie
Universitätsklinikum A.ö.R.
Leipziger Str. 44
39120 Magdeburg

Fax / Aktenzeichen / eMail

Datum: 25.04.2017

Sehr geehrter Herr Kollege PD Piatek,

bei Ihrer Anfrage vom März / April 2017 zur Dissertation von Herrn Peter Hans-Jörg Kurz :
"Weltweite Unterstützung des lokalen Notrufs durch die Übermittlung von Positionsdaten bei der Benutzung von Smartphones"
handelt es sich um die Erfassung und Auswertung von Daten. Diese Daten stammen nicht aus einem Arzt-Patienten-Verhältnis. Auch sind Zuständigkeiten des AMG und des MPG nicht herleitbar.
Gemäß § 15 (1) Berufsordnung der Landesärztekammer Sachsen-Anhalt wird wie folgt formuliert:
„Ärzte, die sich an einem Forschungsvorhaben beteiligen, bei dem in die psychische und körperliche Integrität eines Menschen eingegriffen oder Körpermaterialien oder Daten verwendet werden, ... müssen sicherstellen, dass vor der Durchführung des Forschungsvorhabens eine Beratung erfolgt..."
Die Daten der o. g. Arbeit wurden mit Hilfe von Probanden, die freiwillig an dieser Erhebung teilnahmen, gesammelt, im Konkreten ging es um Ortungsdaten mittels Smartphone. Persönliche Daten, d. h. Messwerte von Flüssigkeiten im Körper, Feststellungen von psychischen Zuständen etc., wurden nicht erhoben, so dass sich medizinethische Ansatzpunkte nach Rücksprache mit dem Kommissionsvorsitzenden nicht zwangsläufig ableiten ließen.

Aus diesen Aspekten heraus nehmen wir Ihre Informationen vom März / April 2017 zur Kenntnis. Die Unterlagen werden unter „Allgemeines 2017" bei uns archiviert, ohne dass ein separater Vorgang angelegt wird.

Mit freundlichen Grüßen

Dr. med. Norbert Beck
Geschäftsführer der Ethikkommission
