# Supplementary material for: Significant acceleration of emergency response using smartphone geolocation data and a worldwide emergency call support system
Source: PLoS One. 2018 May 23;13(5):e0196336. doi: 10.1371/journal.pone.0196336 (PMC5965832; doi:10.1371/journal.pone.0196336)
Supplement: S2 Text — (PDF) [file pone.0196336.s005.pdf]

UNIVERSITÄTSKLINIKUM
MAGDEBURG A.ö.R.

OTTO VON GUERICKE
UNIVERSITÄT
MAGDEBURG

Magdeburg, 25.4.2017

Dear Dr. Piatek,

concerning your request of March / April 2017, the dissertation of Mr. Peter Hans-Jörg Kurz:

"Worldwide support of the local emergency call by the transmission of position data the use of smartphones“

is about the collection and evaluation of data. These data does not emerge from a doctor-patient relationship. In addition, responsibilities of the AMG and the MPG cannot be deduced.

According to § 15 (1) Professional Code of the State Medical Association of Saxony-Anhalt, the following is formulated:

"Doctors who participate in a research project in which psychic and physical Integrity of a person intervened or body materials or data are used ... must ensure that advice is given before the research project is carried out ... "

The data of the above named work was done with the help of volunteers who volunteered for this survey. In particular, it was about location data using a smartphone. Personal data, like measurements of fluids in the body, findings of mental states, etc., were not collected, so that medical ethical approaches after consultation with the Commission chairmen did not necessarily derive.

From these aspects we take note of your information from March I April 2017. The documents are archived under "General 2017“, without creating a separate process.

Best regards

Dr. med. Norbert Beck
Managing Director of the Ethics Committee
